# Robust and Persistent B- and T-Cell Responses after COVID-19 in Immunocompetent and Solid Organ Transplant Recipient Patients

**DOI:** 10.3390/v13112261

**Published:** 2021-11-11

**Authors:** Federica Zavaglio, Vanessa Frangipane, Monica Morosini, Elisa Gabanti, Paola Zelini, Josè Camilla Sammartino, Alessandro Ferrari, Marilena Gregorini, Teresa Rampino, Annalia Asti, Elena Seminari, Angela Di Matteo, Barbara Cattadori, Carlo Pellegrini, Stelvio Tonello, Venkata Ramana Mallela, Rosalba Minisini, Manuela Rizzi, Pier Paolo Sainaghi, Federica Meloni, Daniele Lilleri, Fausto Baldanti

**Affiliations:** 1 Unit of Microbiology and Virology, IRCCS Policlinico San Matteo Foundation, 27100 Pavia, Italy; fede.zavaglio90@gmail.com (F.Z.); e.gabanti@smatteo.pv.it (E.G.); jose.sammartino@iusspavia.it (J.C.S.); alessandro.ferrari04@universitadipavia.it (A.F.); f.baldanti@smatteo.pv.it (F.B.); 2 Research Laboratory of Lung Diseases, Section of Cell Biology, IRCCS Policlinico San Matteo Foundation, 27100 Pavia, Italy; frangipanevanessa@gmail.com (V.F.); m.morosini@smatteo.pv.it (M.M.); f.meloni@smatteo.pv.it (F.M.); 3 Obstetrics and Gynecology, IRCCS Policlinico San Matteo Foundation, 27100 Pavia, Italy; p.zelini@smatteo.pv.it; 4 Unit of Nephrology, Dialysis and Transplantation, IRCCS Policlinico San Matteo Foundation, 27100 Pavia, Italy; m.gregorini@smatteo.pv.it (M.G.); t.rampino@smatteo.pv.it (T.R.); annalia.asti@unipv.it (A.A.); 5 Infectious Diseases Clinic, University of Pavia and IRCCS Policlinico San Matteo Foundation, 27100 Pavia, Italy; e.seminari@smatteo.pv.it (E.S.); a.dimatteo@smatteo.pv.it (A.D.M.); 6 Cardiac Surgery, Department of Intensive Medicine, IRCCS Policlinico San Matteo Foundation, 27100 Pavia, Italy; b.cattadori@smatteo.pv.it (B.C.); c.pellegrini@smatteo.pv.it (C.P.); 7 Department of Clinical, Surgical, Diagnostic and Pediatric Sciences, University of Pavia, 27100 Pavia, Italy; 8 Immunoreumatology Laboratory, Center for Translational Research on Autoimmune and Allergic Disease-CAAD, University of Piemonte Orientale, 28100 Novara, Italy; stelvio.tonello@med.uniupo.it (S.T.); pierpaolo.sainaghi@med.uniupo.it (P.P.S.); 9 Internal Medicine Laboratory, Department of Translational Medicine, University of Piemonte Orientale, 28100 Novara, Italy; rosalba.minisini@med.uniupo.it; 10 Immunorheumatology Unit, Division of Internal Medicine, “Maggiore della Carità” Univerisity Hospital, 28100 Novara, Italy; vramana6565@gmail.com (V.R.M.); manuela.rizzi@med.uniupo.it (M.R.)

**Keywords:** SARS-CoV-2, COVID-19, immunocompetent patients, transplanted patients, spike protein, membrane protein, nucleocapsid protein, antibody response, T-cell response, cytokines

## Abstract

The development and persistence of SARS-CoV-2-specific immune response in immunocompetent (IC) and immunocompromised patients is crucial for long-term protection. Immune response to SARS-CoV-2 infection was analysed in 57 IC and 15 solid organ transplanted (TX) patients. Antibody responses were determined by ELISA and neutralization assay. T-cell response was determined by stimulation with peptide pools of the Spike, Envelope, Membrane, and Nucleocapsid proteins with a 20-h Activation Induced Marker (AIM) and 7-day lymphoproliferative assays. Antibody response was detected at similar levels in IC and TX patients. Anti-Spike IgG, IgA and neutralizing antibodies persisted for at least one year, while anti-Nucleocapsid IgG declined earlier. Patients with pneumonia developed higher antibody levels than patients with mild symptoms. Similarly, both rapid and proliferative T-cell responses were detected within the first two months after infection at comparable levels in IC and TX patients, and were higher in patients with pneumonia. T-cell response persisted for at least one year in both IC and TX patients. Spike, Membrane, and Nucleocapsid proteins elicited the major CD4^+^ and CD8^+^ T-cell responses, whereas the T-cell response to Envelope protein was negligible. After SARS-CoV-2 infection, antibody and T-cell responses develop rapidly and persist over time in both immunocompetent and transplanted patients.

## 1. Introduction

A novel coronavirus named SARS-CoV-2 has been identified as the causative agent of a global outbreak of a respiratory tract disease, referred to as COVID-19 [1,2].

COVID-19 is characterised by fever, cough, dyspnoea, and myalgia. In some patients the infection results in mild symptoms that do not require hospitalization, but pneumonia symptoms that may require invasive mechanical ventilation for a period of several weeks can also occur [2,3].

Several studies reported that IgG antibodies persist longer in immunocompetent patients with severe SARS-CoV-2 infection compared to milder cases [4,5]. According to some studies, the IgG and IgA titres were higher in patients with severe symptoms [5,6,7]. Conversely, a study reported no difference between mild and severe immunocompetent patients [8]. Higher titres of neutralizing antibodies (Nt Ab) were detected in the most clinically severe cases [9,10,11,12,13,14], while no neutralizing activity was detected in plasma from the majority of asymptomatic cases [12].

SARS-CoV-2 Spike (S) protein reactive T cells were identified in immunocompetent patients suffering from moderate, severe, and critical COVID-19 [15] and a dominance of CD4^+^ T-cell over CD8^+^ T-cell response was observed in severe COVID-19 patients [16].

Strong CD4^+^ T-cell reactivity to the viral S, Membrane (M), and Nucleocapsid (N) proteins was observed in mild COVID-19 immunocompetent patients, but M protein induced the highest frequencies of CD4^+^ T cells, when compared to S and N proteins, in severe COVID-19 patients [17].

An important issue is the duration of the immune response. A recent study reported that a T-cell response was measurable in 95% of subjects 5 to 8 months post symptoms, indicating that durable immunity against secondary COVID-19 is possible in immunocompetent patients [18].

However, the characteristics of the immune response to SARS-CoV-2 in immunocompromised patients, such as transplant recipients, has been poorly investigated. A first study analysing the anti-SARS-CoV-2 N IgG antibody in liver transplanted patients showed an earlier and more pronounced decline of IgG serum levels in transplant recipients compared with immunocompetent controls, although anti-N IgG antibody was still detectable 6 months after symptoms onset in most patients [19]. Another study showed no difference in humoral and cellular antiviral immunity between transplanted and non-immunosuppressed patients [20].

The objective of the current study was to evaluate the antigen-specific antibody and T-cell responses in SARS-CoV-2-infected immunocompetent and solid organ transplanted (kidney, lung, and heart) patients with pneumonia or mild symptoms, analysed in the convalescent phase until one year after SARS-CoV-2 infection.

## 2. Materials and Methods

### 2.1. Study Subjects

From March 2020 to December 2020, 72 post-COVID-19 patients (57 immunocompetent (IC) and 15 solid organ transplanted (TX) patients) were enrolled in the study after diagnosis of SARS-CoV-2 infection by nasal swab testing. TX patients were receiving immunosuppressive treatment with a calcineurin inhibitor plus mofetil-mycophenolate (*n* = 10) or everolimus (*n* = 4), and one patient was receiving sirolimus plus mofetil-mycophenolate. In addition, four patients were receiving low dose steroid treatment. The study protocol was approved by the ethics committee (P-20200046007) and patients signed informed consent.

Blood samples from 30 IC patients with pneumonia were collected in the convalescent phase of the infection, after viral clearance (median: 58; range (45–100) days after infection) and 11 of them were analysed also at a late time point (212; (186–400) days). In addition, 14 IC patients with mild symptoms were analysed at the early time point (48; (30–100) days) and 13 other IC patients with mild symptoms were analysed at a late time point (192; (150–306) days).

Among TX patients, 9 had pneumonia and 6 mild symptoms; sequential blood samples from TX patients were collected at sequential time points (from 5 to 309 days) after infection. For comparison with IC patients, we selected an early (patients with pneumonia: 60; (30–62) days, and patients with mild symptoms: 54; (26–75) days) and a late time point (patients with pneumonia: 233; (164–309) days, and patients with mild symptoms: 167; (150–207) days) after infection. 

Pneumonia was defined on the basis of a chest x-ray. The main clinical characteristics of the patients are shown in Table 1.

### 2.2. Antibody Assays

Anti-S IgA and IgG, and anti-N IgG were determined by ELISA (Euroimmun AG, Luebeck, Germany) according to the manufacture’s guidelines. Results were evaluated semi-quantitatively by calculation of the ratio of the extinction of the control or patient sample over the extinction of the calibrator. This ratio was interpreted as follows: <0.8 negative; ≥0.8 to <1.1 borderline; ≥1.1 positive.

Neutralizing antibody (Nt Ab) serum titre was determined as previously reported [21]. Results were considered positive if higher or equal to 1:10 serum titre.

### 2.3. Protein Peptide Pools

To evaluate the antigen-specific T-cellular response, peptide pools (15 mers, overlapping by 10 amino acids, Pepscan, Lelystad, The Netherlands) representative of the S, Envelope (E), M, and N proteins, were used. A peptide pool of human actin (15 mers, overlapping by 10 amino acids, Pepscan, Lelystad, The Netherlands) was used as a negative control.

### 2.4. PBMC Isolation

Peripheral whole blood was collected in serum separator tubes and heparin-treated tubes. Peripheral blood mononuclear cells (PBMCs) were isolated by standard density gradient centrifugation using Lymphoprep (Sentinel Diagnostics, Milano, Italy). Isolated PBMCs were cryopreserved in cell freezing medium containing 10% dimethyl sulfoxide (DMSO) (Corning, NY, US.), supplemented with 90% heat inactivated fetal bovine serum (FBS, Sigma, St. Louis, MO, US) and stored in liquid nitrogen. 

### 2.5. Activation Induction Marker Assay

To evaluate antigen-specific rapid T-cell response, PBMCs were stimulated for 20 h with SARS-CoV-2 specific peptide pools from S, E, M, N, and peptide pool of human actin [1 µg/mL] in the presence of co-stimulator molecules CD28 and CD49d (BD Bioscience, Franklin Lakes, New Jersey, USA. Cell were seeded in 96-wells round bottom plates at a density of 0.5–1 × 10^6^ cells/200 µL culture medium per well. Culture medium was RPMI 1640 (Euroclone, Milano, Italy) supplemented with 2 mM L-glutamine (Euroclone), 100 U/mL penicillin and 100 µg/mL streptomycin solution (Euroclone), and 10% of heat inactivated FBS. 

After culture, cells were washed with PBS 2 mM EDTA and stained in PBS with Live/Dead Fixable Violet Dye (Invitrogen, Waltham, Massachusetts, USA) for 30 min at 4 °C. After rising with PBS and staining in PBS 5% FCS with CD4 APC Cy7 (BD Biosciences), CD8 V500 (BD Biosciences), CD25 PECy7 (BD Biosciences) and CD137 PECy5 (BD Biosciences) antibodies for 30 min at 4 °C. Finally, cells were washed and resuspended in PBS 1% paraformaldehyde. 

Antigen-specific T-cell frequency was determined by subtracting the frequency of CD25^+^CD137^+^ CD4^+^ or CD8^+^ detected in PBMC incubated with actin peptides from the frequency of CD25^+^CD137^+^ CD4^+^ or CD8^+^ detected in PBMC incubated with SARS-CoV-2 peptides. To determine the cut-off for antigen-specific T-cell frequency, samples from seven SARS-CoV-2-seronegative subjects were tested. A value < 0.05% antigen-specific T-cells was considered negative while a value ≥ 0.05% was considered positive.

Flow cytometry analyses were performed with a FACS Canto II flow cytometer and BD DIVA software (BD Biosciences). A representative pseudocolor plot analysis is shown in Supplementary Figure S1.

### 2.6. Antigen-Specific Cytokine Production

Supernatant concentrations of cytokines and chemokines were measured in duplicate using BioPlex Pro Human Cytokine Screening Panel (27-Plex #M500KCAF0Y, Bio-Rad, Hercules, CA, United States) according to the manufacturer’s instructions. All the cells’ supernatants were inactivated for 60 min at 56 °C at the same time before analysis. The supernatants were measured undiluted and culture medium was used as diluent for standards and control. All the results are analysed with BIO-PLEX manager software 6.0.

### 2.7. Detection of Antigen-Specific CD4^+^and CD8^+^ T-Cell Proliferative Response

To evaluate antigen-specific proliferative response, PBMCs (600,000/200μL culture medium per well) were stimulated in triplicate in 96-well round-bottom plates with SARS-CoV-2 and human actin peptide pools at a final concentration of 0.1 µg/mL for 7 days. Culture medium was the same as the AIM assay supplemented with 5% heat inactivated human serum AB (Sigma), 1 mM Sodium Pyruvate (Gibco, Grand Island, NY, USA), 100 µM non-essential amino acids (Euroclone), and 50 µM 2-Mercaptoethanol (Gibco). After culture, cells were washed, stained with Live/Dead Fixable Violet Dye (Invitrogen) and subsequently with CD3 PerCP 5.5 (BD), CD4 APC Cy7 (BD), CD8 FITC (BD), CD25 PECy7 (BD), CD278 (ICOS) APC (Invitrogen). Finally, cells were washed and resuspended in PBS 1% paraformaldehyde.

A Cell Proliferation Index (CPI) for antigen-specific expanded T-cells was determined by subtracting the percentage of CD25^+^ICOS^+^ CD3^+^CD4^+^ or CD3^+^CD8^+^ detected in PBMC incubated with actin peptides from the percentage of CD25^+^ICOS^+^ T-cell subsets detected in PBMC incubated with SARS-CoV-2 peptides. To determine the cut-off for antigen-specific CPI, samples from five SARS-CoV-2-seronegative subjects were tested. A CPI <1.5% was considered negative while a value ≥1.5% was considered positive.

Flow cytometry analyses were performed with a FACS Canto II flow cytometer and BD DIVA software (BD Biosciences). A representative pseudocolor plot analysis is shown in Supplementary Figure S2.

### 2.8. Statistical Analysis

Statistical analyses were performed with GraphPad Prism 6. The Mann–Whitney U-test or Wilcoxon signed-rank test were applied for unpaired or paired comparison, respectively, while the Friedman test was applied for multiple comparisons.

## 3. Results

### 3.1. Characteristics of the Patients Analysed

As reported in Table 1, IC patients with pneumonia were significantly older than patients with mild symptoms, whereas no difference in age was observed between TX patients with pneumonia or mild symptoms. IC patients with pneumonia required a higher oxygen supply than TX patients, whereas durations of SARS-CoV-2 infection (i.e., duration of SARS-CoV-2 RNA positivity in nasopharyngeal swabs) was not significantly different among the groups of patients. One of the six TX patients with mild symptoms died from sudden death five months after the resolution of SARS-CoV-2 infection, with no apparent direct link with COVID-19. 

### 3.2. Antigen-Specific Antibody Response in Convalescent COVID-19 IC and TX Patients

**Figure 1:**
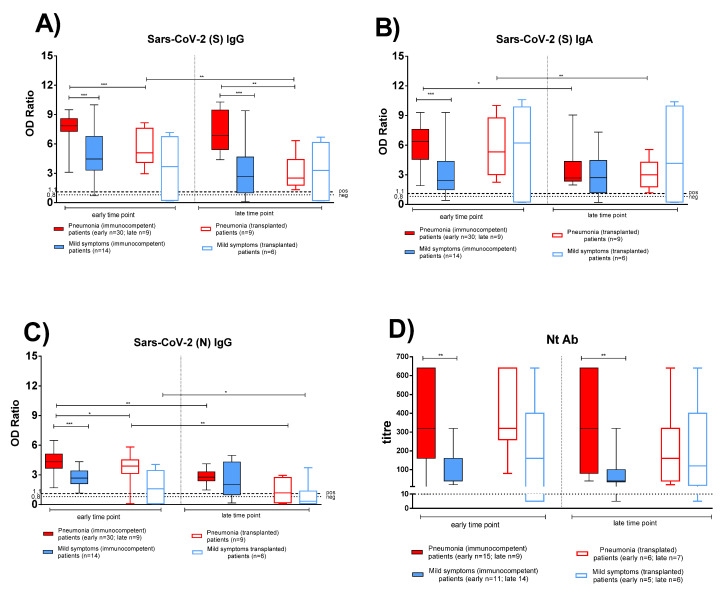
The antigen-specific antibody response was compared between immunocompetent (IC) and transplanted (TX) patients with pneumonia or mild symptoms at early and late time points. (**A**) Anti-Spike (S) IgG. (**B**) Anti-Spike (S) IgA. (**C**) Anti-Nucleocapsid (N) IgG. (**D**) Nt Ab. Early time point: patients with pneumonia, IC median 58 (range 45–100); TX 60 (30–62) days after infection; patients with mild symptoms, IC 48 (30–100); TX 54 (26–75) days after infection. Late time point: patients with pneumonia, IC median 212 (range 186–400); TX 233 (164–309) days after infection; patients with mild symptoms, IC 192 (150–306); TX 167 (150–207) days after infection. * *p* < 0.05, ** *p* < 0.01, *** *p* < 0.001.

SARS-CoV-2 specific antibody response in post COVID-19 IC and TX patients was compared at two sequential time points during convalescent periods, in both pneumonia and mild symptoms patients. All subjects with pneumonia showed detectable anti-S IgG and IgA from two months until one year after infection (Figure 1A,B). Notably, higher antibody levels developed in IC patients with pneumonia (Figure 1 solid red bars) than in IC patients with mild symptoms (Figure 1 solid blue bars). Anti-S IgG was higher in IC patients with pneumonia both at early (*p* < 0.001) and late (*p* < 0.001) time points, whereas anti-S IgA and anti-N IgG were higher in IC patients with pneumonia only at the early time point. In TX patients, no significant difference was observed for anti-S IgG, anti-S IgA, and anti-N IgG between patients with pneumonia (Figure 1 empty red bars) and patients with mild symptoms (Figure 1 empty blue bars). Only two TX patients, who had gastrointestinal symptoms only, did not develop a detectable antibody response.

Peak antibody response was detected within the first two months after infection and a significantly higher level was detected in IC than in TX patients with pneumonia for anti-S and anti-N IgG (*p* < 0.001 and *p* = 0.030, respectively; Figure 1A,C). In IC patients with pneumonia, while anti-S IgG persisted at constant levels for at least one year after infection, a decline of anti-N IgG and anti-S IgA levels (*p* = 0.007 and *p* = 0.019, respectively) was observed. The three antibody subclasses declined at late time points in TX patients with pneumonia, but did not change significantly in both IC and TX patients with mild symptoms, (only 2/13 IC patients with mild symptoms did not display anti-S and anti-N antibodies at the late time point).

A trend similar to that of anti-S IgG was observed for Nt Abs. Nt titre was higher in IC patients with pneumonia than in patients with mild symptoms at early (*p* = 0.060) and late (*p* = 0.048) time points (Figure 1D). In TX patients, no significant difference was observed in Nt Ab levels in patients with pneumonia and mild symptoms at early and late time points (Figure 1D). Nt Abs persisted for at least one year in most IC and TX patients.

**Figure 2:**
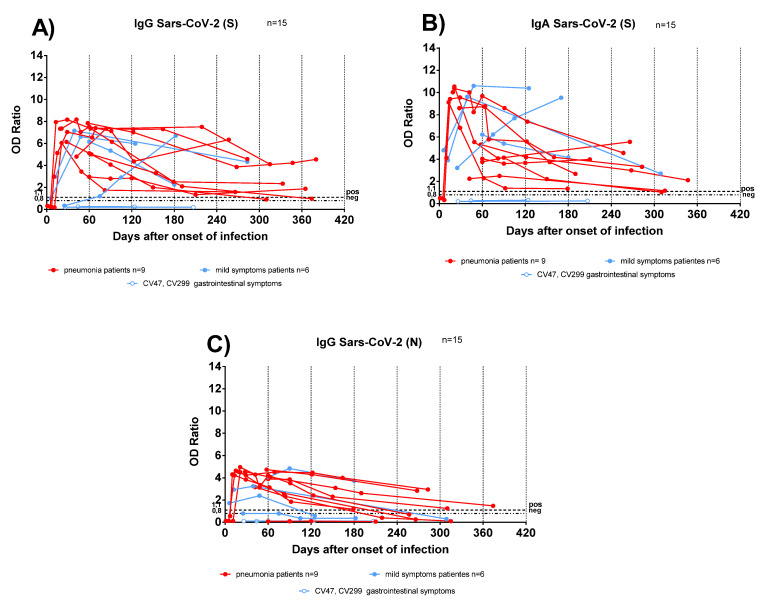
Antibody response kinetics in transplanted patients with pneumonia or mild symptoms. (**A**) Anti-Spike (S) IgG; (**B**) anti-Spike (S) IgA; (**C**) anti-Nucleocapsid (N) IgG. Red lines represent pneumonia patients; light blue lines represent patients with mild symptoms. Light blue lines with white circles represent patients with gastrointestinal symptoms.

Antibody responses were analysed in sequential samples from TX patients: anti-S IgG and IgA persisted for at least one year after infection (Figure 2A,B), conversely anti-N IgG started to decline as early as 90 days after COVID-19 diagnosis (Figure 2C). In two TX patients with mild symptoms, the antibody levels increased over time. We could hypothesize that sustained virus replication below detection levels, or an undiagnosed secondary infection, might have boosted the antibody response.

### 3.3. Effector and Memory T-Cell Responses in IC and TX Patients after COVID-19

**Figure 3:**
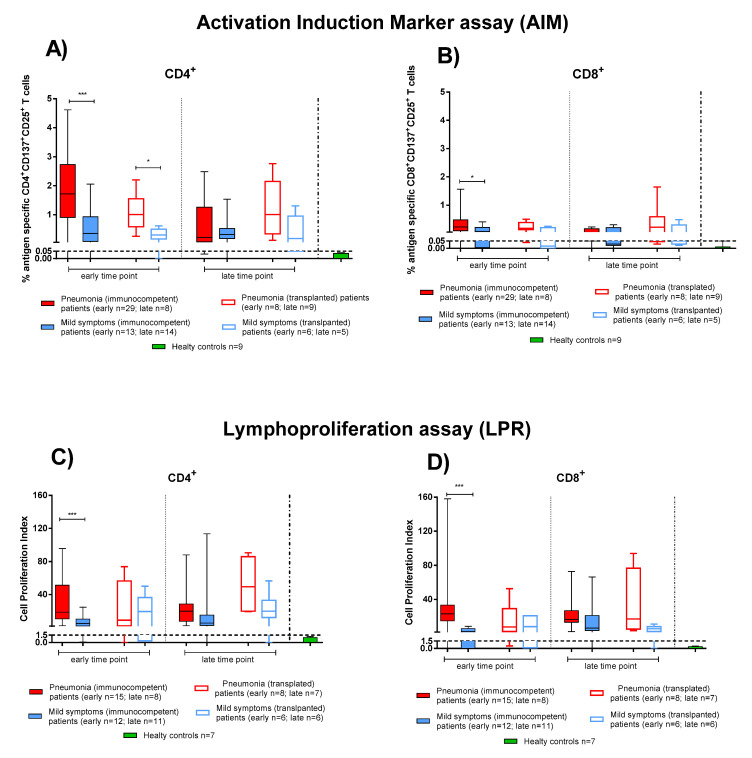
SARS-CoV-2-specific T-cell responses were compared between immunocompetent (IC) and transplanted (TX) patients with pneumonia or mild symptoms at early and late time points. (**A**) SARS-CoV-2-specific CD4^+^ and (**B**) CD8^+^ T-cells (rapid Activation Induced Marker assay); (**C**) CD4^+^ T-Cell Proliferation Index; (**D**) CD8^+^ T-Cell Proliferation Index. Early time point: patients with pneumonia, IC median 58 (range 45–100); TX 60 (30–62) days after infection; patients with mild symptoms, IC 48 (30–100); TX 54 (26–75) days after infection. Late time point: patients with pneumonia, IC median 212 (range 186–400); TX 233 (164–309) days after infection; patients with mild symptoms, IC 192 (150–306); TX 167 (150–207) days after infection. * *p* < 0.05; *** *p* < 0.001.

We considered the SARS-CoV-2 T-cell response as the sum of the single antigen-specific responses. Within the first two months after COVID-19 onset, both IC and TX patients showed SARS-CoV-2 specific rapid T-cell response (as detected by the AIM assay) at similar levels. At the early time point, CD4^+^ T-cell levels were higher in patients with pneumonia (Figure 3 IC solid red bars, TX empty red bars) than those with mild symptoms (Figure 3 IC solid blue bars, TX empty blue bars) (in IC *p* < 0.001 and in TX *p* = 0.013) (Figure 3A). A similar difference was also observed at early time point for CD8^+^ T cells (in IC *p* = 0.016), although SARS-CoV-2 CD8^+^ T-cell levels were lower than CD4^+^ (Figure 3B). 

We also evaluated the T-cell proliferative response in IC and TX patients with pneumonia and mild symptoms after SARS-CoV-2 infection. Only in IC patients at the early time point did we observe a significant difference between patients with pneumonia or mild symptoms in proliferative CD4^+^ and CD8^+^ T-cell responses (*p* < 0.001). Nevertheless, as also observed for rapid T-cell activation, SARS-CoV-2-specific T-cell proliferation was detected mainly in the CD4^+^ than the CD8^+^ T-cell subset (Figure 3C,D). Interestingly, the antigen specific T-cell proliferative response did not decrease significantly until the late time point (Figure 3) in both IC and TX patients. 

The production of Th1 (IFNγ, TNFα, IL-2, MIP-1α and MIP-1β) and Th2 (IL-4 and IL-5) cytokines and IL-10 was evaluated after 20 h stimulation with S protein in IC and TX patients.

**Figure 4:**
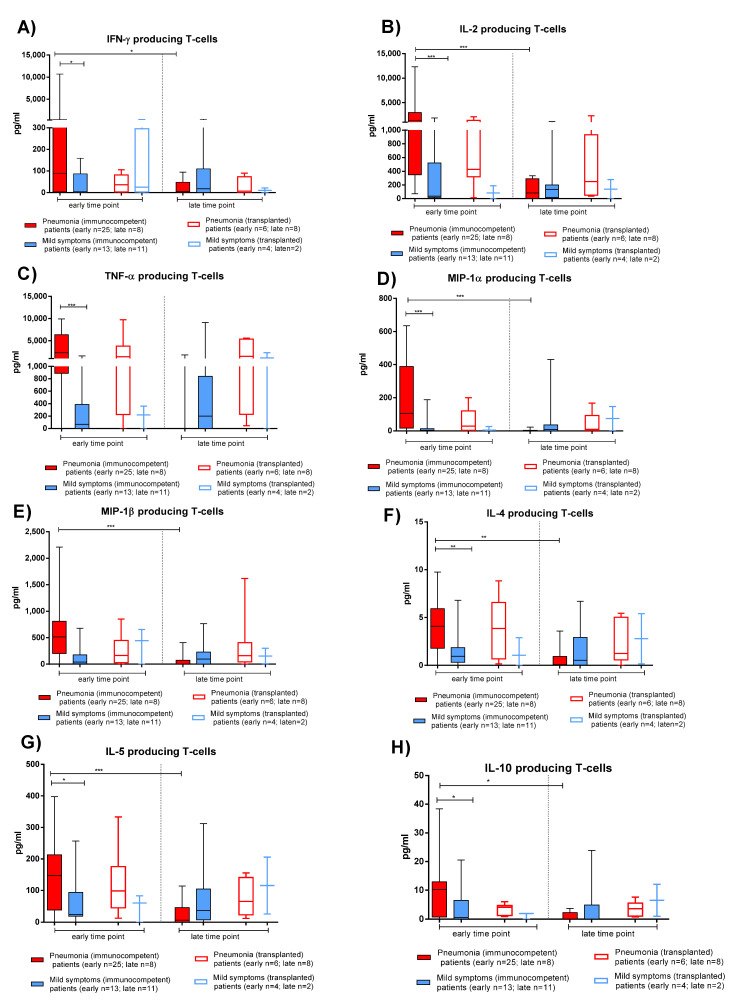
Cytokine production of T-cells after stimulation with Spike (S) peptide pools in immunocompetent and transplanted patients with pneumonia or mild symptoms at early and late time points. (**A**) IFN-γ. (**B**) IL-2. (**C**) TNF-α. (**D**) MIP-1α. (**E**) MIP-1β. (**F**) IL-4. (**G**) IL-5. (**H**) IL-10. Early time point: patients with pneumonia, median 59 (range 30–100) days after infection; patients with mild symptoms, 52 (26–100) days after infection. Late time point: patients with pneumonia, median 220 (range 164–400); days after infection; patients with mild symptoms, 189 (150–306) days after infection. * *p* < 0.05; ** *p* < 0.01; *** *p* < 0.001.

As observed with the AIM assay, PBMCs from IC patients with pneumonia produced the higher levels of both Th1 and Th2 cytokines at the early time point (Figure 4, solid red bars). A significant decrease of cytokine production in IC patients with pneumonia was observed at the late time point. Similar levels of cytokine production were observed in TX patients with pneumonia (Figure 4, empty red bars) or mild symptoms (Figure 4, empty blue bars). There was no difference in the relative production of Th1 and Th2 cytokines between patients with pneumonia or mild symptoms. In both the IC and TX groups, IL-10 production was detected at low levels, with higher concentrations detected in IC patients with pneumonia.

**Figure 5:**
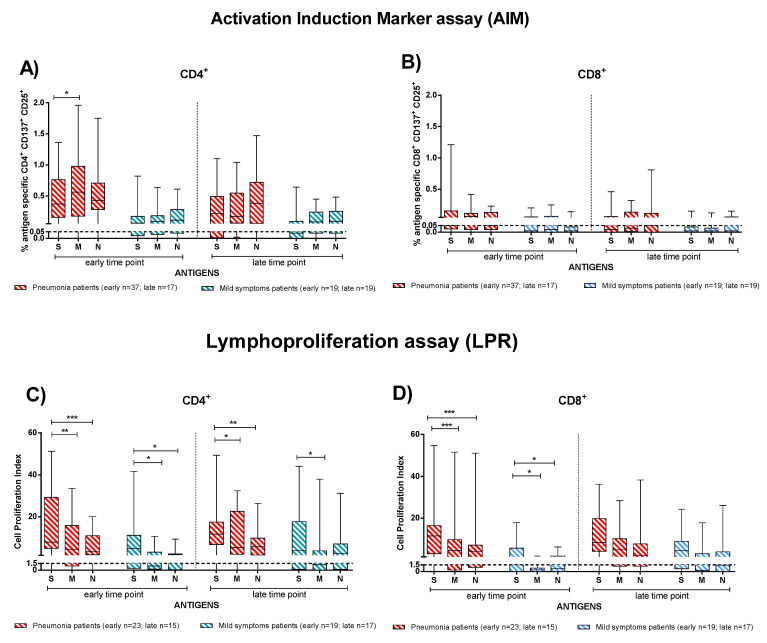
The T-cell dominant specificity after stimulation with Spike (S), Membrane (M), and Nucleocapsid (N) peptide pools in patients with pneumonia or mild symptoms at early and late time points (immunocompetent and transplanted were considered together). (**A**) Antigen-specific CD4^+^ T-cells and (**B**) CD8^+^ T-cells (rapid Activation Induced Marker assay); (**C**) CD4^+^ T-Cell Proliferation Index; (**D**) CD8^+^ T-Cell Proliferation Index. Early time point: patients with pneumonia, median 59 (range 30–100) days after infection; patients with mild symptoms, 52 (26–100) days after infection. Late time point: patients with pneumonia, median 220 (range 164–400); days after infection; patients with mild symptoms, 189 (150–306) days after infection. * *p* < 0.05; ** *p* < 0.01; *** *p* < 0.001.

In order to investigate which of the SARS-CoV-2 antigens (S, M, and N proteins) elicited the major T-cell response, we considered IC and TX patients together and analysed the T-cell response by dividing the subjects into patients with pneumonia (Figure 5, red bars) and mild symptoms (Figure 5, blue bars). T-cell response to E protein was negligible. At the early time point, pneumonia patients showed a slightly higher rapid CD4^+^ T-cell response directed more against the M than S protein (*p* = 0.020), but no difference was observed at the late time point (Figure 5A). No significant difference in antigen specificity was observed in the CD8^+^ T-cell response (Figure 5B) and in patients with mild symptoms.

Conversely, we observed that S protein induced the major CD4^+^ and CD8^+^ T-cell proliferative response (Figure 5C,D). In particular, we noticed higher response to S than N (*p* < 0.001 for CD4^+^ and CD8^+^) or M proteins (*p* = 0,040 for CD4^+^and *p* < 0.001 CD8^+^) in the pneumonia group at the early time point. Additionally, in mildly symptomatic patients, CD4^+^ and CD8^+^ T-cells proliferated in response to the S protein significantly more than to the M protein (*p* = 0.020) at the early time point post infection (Figure 5C,D). At the late time point, a similar trend was observed for the CD4^+^ T-cell proliferative response particularly in pneumonia patients. We demonstrate the antigen-specific T-cell proliferative response separately considering the immunocompetent and transplanted patients in Supplementary Figure S3.

According to our observation, the antigen specific T-cell response was better detected with the proliferation than the AIM assay at the late time point. In fact, 5/31 patients did not show SARS-CoV-2 specific CD4^+^ T-cells with the AIM assay, while a proliferative response was still detectable. Similarly, 13/31 patients with no CD8^+^ response according to the AIM assay showed a proliferative response. 

## 4. Discussion

In this study, we evaluated the antigen-specific antibody and T-cell responses in post-COVID-19 IC and TX patients with pneumonia or mild symptoms, analysed from the convalescent phase until one year after infection. 

Here, we show that all patients with pneumonia and most patients with mild symptoms (both IC and TX) developed detectable and persistent anti-S IgG and IgA antibodies from two months until at least one year after infection (although antibody levels decreased with time), while anti-N IgG levels disappeared at the late time point in most TX patients. Notably, IC but not TX patients with pneumonia developed higher IgG, IgA, and Nt Ab levels than patients with mild symptoms. Only two TX patients, who had isolated gastrointestinal symptoms, did not develop a detectable antibody response. We could hypothesize that in these two patients the viral load was not sufficient to elicit a systemic antibody response, but triggered an immune reaction at the local mucosal level only. At the early time point, rapid CD4^+^ T-cell response was higher in patients with pneumonia, while the CD8^+^ T-cell response was poor in all patients. We also observed a significant difference at the early time point in proliferative CD4^+^ and CD8^+^ T-cell responses between IC (but not TX) patients with pneumonia or mild symptoms. However, conversely to antibody levels, antigen specific T-cell responses did not decrease significantly within 12 months after infection both in IC and TX patients. 

Interestingly, both the Nt Ab and T-cell responses developed at similar levels in IC and TX patients, although, among patients with pneumonia, we observed a higher anti-S and anti-N IgG antibody levels in IC patients. This observation could be explained by the fact that, according to the oxygen supply required, the IC patients analysed in our study had more severe pneumonia than the TX patients. Therefore, we could hypothesize that IC pneumonia patients received a higher inflammatory stimulus, which may have contributed to the induction of a higher but not more functional (since the Nt Ab response was not significantly different) IgG antibody response. Although the levels of antibodies were sustained overtime especially in pneumonia patients, at present the level required for protection against secondary infections or disease is unknown.

Regarding the antigen specificity of the T-cell response, while T-cells elicited by the E protein were negligible, we did not find a significant difference in the rapid response for the S, M, or N proteins (with the exception of the higher CD4^+^ T-cell response for the M protein in IC patients with pneumonia at the early time point). Conversely, we observed that the S protein induced the major proliferative CD4^+^ and CD8^+^ T-cell responses. We observed no difference in Th1 and Th2 cytokines production in IC and TX patients, except that IC patients with pneumonia produced the higher levels of cytokines. This is in line with the higher rapid T-cell activation observed in this group of patients with the AIM assay. Previous reports reported Th2-skewed cytokine production in SARS-CoV infected patients developing pneumonia, but no difference was found in the quality of cytokines detected in S-specific T-cells in patients developing pneumonia after SARS-CoV-2 infection [22].

So far, few studies investigated antibody and T-cell immunity in COVID-19 TX patients [19,21]. However, these studies have exclusively focused on the acute infection and convalescent phase, while reports on long-term persistence of immunity are missing.

Previous studies showed that TX patients achieve a serological response and T-cell cytokine production comparable to that of IC patients during the early convalescent phase. Nonetheless, a certain delay in achieving such strong immune response was observed among TX patients [23]. In addition, liver transplanted patients exhibited lower persistence of anti-N IgG antibodies within the first 6 months post-infection and a more pronounced antibody levels decline [19], as also observed in our study.

Several studies reported that IgG and IgA levels were higher in IC patients with severe symptoms [5,6,7], while other studies reported no difference between mild and severe IC patients [8]. Our data confirm that IC but not TX patients with pneumonia (the latter showing less severe disease) develop higher levels of anti-S and anti-N IgG, and anti-S IgA than patients with mild symptoms. 

We observed a dominance of rapid CD4^+^ over CD8^+^ T-cell response in post-COVID-19 IC (and TX) patients, as reported by Weiskopf et al. [16]. Concerning the antigen specificity of the T-cell response, strong reactivity to the viral S and M proteins in IC patients with mild and severe symptoms was reported [17]. We observed a slightly major rapid CD4^+^ T-cell response directed to the M protein only in IC pneumonia patients and we can support that the “effector” T-cell response appears the same for all SARS-CoV-2 proteins analysed (S, M and N), while the major reactivity of the proliferative T-cell response is directed towards the S protein. In addition, the proliferative assay appears more sensitive in detecting the CD4^+^ and, particularly, CD8^+^ T-cell responses compared to the AIM assay, especially at the late time point.

Dan et al. reported a duration of the immune response until at least 5–8 months post symptoms [18]; we extended the observation of the persistence of the immune response until one year after infection. 

Different studies have reported a significantly higher risk of fatal outcomes among TX patients developing COVID-19 as compared to the healthy population [23,24,25,26,27]. The main hypothesis for these poorer outcomes is based on their T-cell immunocompromised status. Nevertheless, we observed robust and persistent antigen-specific antibody and T-cell responses at comparable levels in TX and IC patients. Although most patients in our study were more than 6 months post-transplantation and in the immunosuppression maintenance phase, we did not observe an impaired immune response in the three patients of the study who developed COVID-19 soon after transplantation. In contrast to trends observed with mRNA SARS-CoV-2 vaccines, which are less effective in eliciting B and T-cell responses in TX than IC individuals [28,29,30], we observed natural SARS-CoV-2 infection elicits a robust immune response in both patient groups. It is possible that natural infection provides stronger or more prolonged stimulation than mRNA vaccines, able to overcome the immunosuppressed status of TX patients, while administration of three instead of two vaccines does appear to be a better strategy for transplant recipients [31,32]. In addition, a previous study conducted at our Institution showed that the outcome of TX patients was substantially favourable after SARS-CoV-2 infection [33]. The low severity of COVID-19 in our cohort of TX patients might be explained both by the rigorous controls adopted in fragile patients, which may have facilitated their early diagnosis, hospitalization, and treatment, and by the fact that their immunosuppression status did not impair the development of an effective immune response. In addition, immunosuppressive therapy may have avoided the development of the immune-mediated inflammatory complications observed in severe COVID-19 patients. The higher oxygen requirement of IC patients might have been due to the excess inflammation and, therefore, damage to tissues, which may have been reduced by immunosuppressive agents in TX patients. However, the number of patients analysed in our study is too low to draw information on the severity of COVID-19 in TX vs. IC patients. 

There are some limitations in this study such as the small sample size and cross-sectional analysis conducted in IC patients with mild symptoms at early and late time points.

In conclusion, despite immunosuppressive therapy, TX patients developed a robust immune response after SARS-CoV-2 infection, comparable to that observed in IC patients. The immune response persists until one year after SARS-CoV-2 infection, and it is likely that the immunological memory could persist for a long time and may protect both the IC and TX populations from SARS-CoV-2 secondary infections [34,35]. Future evaluation of the magnitude and persistence of the immune response elicited by SARS-CoV-2 vaccines in transplant recipients would provide essential information about immune protection from COVID-19 that could be achieved in this fragile population. 

## Figures and Tables

**Table 1:** Patient characteristics.

	Immunocompetent (IC) (n = 57)		*p* Value	Transplanted (TX) (n = 15)		*p* Value	*p* Value IC vs. TX
	Pneumonia	Mild		Pneumonia	Mild		
Subjects % (n)	53% (30)	47% (27)		60% (9)	40% (6)		
Transplanted Organ:							
Kidney % (n)	na	na		78% (7)	33% (2)		
Heart % (n)	na	na		11% (1)	33% (2)		
Lung % (n)	na	na		11% (1)	33% (2)		
Time After Transplant, Median [range] months	na	na		89 [3–288]	20 [1–43]		
Age, Median [range]	62 [44–81]	45 [21–61]	*p* < 0.001	58 [48–71]	59 [39–65]	*p* = 0.556	*p* = 0.119
Sex, M/F % (n)	63% (19)/37% (11)	48% (13)/52% (14)	*p* = 0.358	78% (7)/22% (2)	83% (5)/18% (1)	*p* = 1	*p* = 0.079
Symptoms:							
Fever % (n)	90% (27)	70% (19)		100% (9)	33% (2)		
Rhinitis %(n)	0	24% (8)		0	0		
Cough % (n)	43% (13)	26% (7)		50% (4)	33% (2)		
Sore Throat % (n)	0	11% (3)		13% (1)	0		
Conjunctivitis % (n)	0	0		13% (1)	0		
Ageusia % (n)	7% (2)	56% (15)		0	33% (2)		
Anosmia % (n)	3% (1)	56% (15)		0	0		
Gastrointestinal % (n)	17% (5)	19% (5)		50% (4)	50% (3)		
Headache % (n)	3% (1)	44% (12)		13% (1)	33% (2)		
O2 supply, % patients (n):							
no	3% (1)	100% (27)		56% (5)	100% (6)		
<5 mL/min	27% (8)	0		33% (3)	0		
≥5 mL/min	70% (21)	0		11% (1)	0		*p* < 0.001
Duration of SARS-CoV-2 infection, Median [range] Days	20 [4–38]	20 [12–29]		17 [10–36]	7 [4–25]		*p* = 0.773
Outcome:							
Live % (n)	100% (30)	100% (27)		100% (9)	83% (5)		

na = not available.

## Data Availability

The data that support the findings of this study are available on request from the corresponding author. The data are not publicly available due to privacy or ethical restrictions.

## Supplementary Materials

The following are available online at https://www.mdpi.com/article/10.3390/v13112261/s1, Figure S1. Activation Induction Marker Phenotyping Flow Cytometry. Representative gating of antigen specific effector CD4+ and CD8+ T-cells from post COVID-19 immunocompetent patients PBMC after stimulation with Spike, Membrane, Nucleocapsid and Human actin peptide pools. Briefly, lymphocytes cells were gated out of all events followed by subsequent singlet gating. Live cells are gated as Pacif Blue and cells were then gated as CD4 APC Cy7+ and CD8 V500+. T-cells were further subdivided into CD4+ CD137 PECy5+ CD25 PECy7+ and CD8+ CD137 PECy5+ CD25 PECy7+ population, Figure S2. Lymphoproliferation Phenotyping Flow Cytometry. Representative gating of antigen specific memory CD4+ and CD8+ T-cells from post COVID-19 immunocompetent patients PBMC after stimulation with Spike, Membrane, Nucleocapsid and Human actin peptide pools. Briefly, lymphocytes cells were gated out of all events followed by subsequent singlet gating. Live cells are gated as Pacif Blue and cells were then gated as CD3 PERCP5.5+. T cells were further subdivided into CD4 APC Cy7+ and CD8 FITC+ population. T cells were defined as CD4+ ICOS APC+ CD25 PECy7+ and CD8+ ICOS APC+ CD25 PECy7+, Figure S3. The T-cell dominant specificity after stimulation with Spike (S), Membrane (M) and Nucleocapsid (N) peptide pools in immunocompetent (IC) and transplanted (TX) patients with pneumonia or mild symptoms at early and late time points. (A) SARS-CoV-2-specific CD4+ and (B) CD8+ T-cells (rapid Activation Induced Marker assay); (C) CD4+ T Cell Proliferation Index; (D) CD8+ T-Cell Proliferation Index. Early time point: patients with pneumonia, IC median 58 (range 45-100); TX 60 (30-62) days after infection; patients with mild symptoms, IC 48 (30-100); TX 54 (26-75) days after infection. Late time point: patients with pneumonia, IC median 212 (range 186-400); TX 233 (164-309) days after infection; patients with mild symptoms, IC 192 (150-306); TX 167 (150-207) days after infection. * *p* < 0.05; ** *p* < 0.01; *** *p* < 0.001.

## References

[1] Chan J.F., Yuan S., Kok K.H., Kai-Wang To K., Chu H., Yang J., Xing F., Liu J., Chik-Yan Yip C., Wing-Shan Poon R. (2020). A familial cluster of pneumonia associated with the 2019 novel coronavirus indicating person-to-person transmission: A study of a family cluster. Lancet.

[2] Huang C., Wang Y., Li X., Ren L., Zhao J., Hu Y., Zhang L., Fan G., Xu J., Gu X. (2020). Clinical features of patients infected with 2019 novel coronavirus in Wuhan, China. Lancet.

[3] Chen G., Wu D., Guo W., Cao Y., Huang D., Wang H., Wang T., Zhang X., Chen H., Yu H. (2020). Clinical and immunological features of severe and moderate coronavirus disease 2019. J. Clin. Investig..

[4] Candel González F.J., Viñuela-Prieto J.M., Del Castillo J.G., García P.B., Saavedra M.F., Píriz A.H., Jiménez Virumbrales D., Canora Lebrato J., García de Casasola G., Gil Prieto R. (2020). Utility of lateral flow tests in SARS-CoV-2 infection monitorization. Rev. Esp. Quimioter..

[5] Carsetti R., Zaffina S., Mortari E.P., Terreri S., Corrente F., Capponi C., Palomba P., Mirabella M., Cascioli S., Palange P. (2020). Different innate and adaptive immune response to SARS-CoV-2 infection of asymptomatic, mild and severe cases. Front. Immunol..

[6] Borremans B., Gamble A., Prager K., Helman S., McClain A., Cox C., Savage V., Lloyd-Smith J.O. (2020). Quantifying antibody kinetics and RNA shedding during early-phase SARS-CoV-2 infection by time since symptom onset. eLife.

[7] Ou J., Tan M., He H., Tan H., Mai J., Long Y., Jiang X., He Q., Huang Y., Li Y. (2020). Study on the expression levels of antibodies against SARS-CoV-2 at different period of disease and its related factors in 192 cases of COVID-19 patients. MedRxiv.

[8] Qin C., Zhou L., Hu Z., Zhang S., Yang S., Tao Y., Xie C., Ma K., Shang K., Wang W. (2020). Dysregulation of immune response in patients with (COVID-19) in Wuhan, China. Clin. Infect. Dis..

[9] Huang A.T., Garcia-Carreras B., Hitchings M.D.T., Yang B., Katzelnick L., Rattigan S.M., Borgert B.A., Moreno C.A., Solomon B.D., Rodriguez-Barraquer I. (2020). A systematic review of antibody mediated immunity to coronaviruses: Antibody kinetics, correlates of protection, and association of antibody responses with severity of disease. Nat. Commun..

[10] Zhang J., Qu X., Liu Z., Wang Q., Wu J., Hu Y., Bai T., Xie T., Huang M., Wu T. (2021). Spike-specific circulating T follicular helper cell and cross-neutralizing antibody responses in COVID-19 convalescent individuals. Nat. Microbiol..

[11] Fafi-Kremer S., Bruel T., Madec Y., Grant R., Tondeur L., Grzelak L., Staropoli I., Anna F., Souque P., Fernandes-Pellerin S. (2020). Serologic responses to SARSCoV-2 infection among hospital staff with mild disease in eastern France. medRxiv.

[12] Brochot E., Demey B., Touze A., Belouzard S., Dubuisson J., Schmit J.L., Duverlie G., Francois C., Castelain S., Helle F. (2020). Anti-Spike anti-Nucleocapsid and neutralizing antibodies in SARS-CoV-2 inpatients and asymptomatic individuals. Front. Microbiol..

[13] Wang X., Guo X., Xin Q., Pan Y., Hu Y., Li J., Chu Y., Feng Y., Wang Q. (2020). Neutralizing Antibody Responses to Severe Acute Respiratory Syndrome Coronavirus 2 in Coronavirus Disease 2019 Inpatients and Convalescent Patients. Clin. Infect. Dis..

[14] Wang P., Liu L., Nair M.S., Yin M.T., Luo Y., Wang Q., Yuan T., Mori K., Guzman Solis A., Yamashita M. (2020). SARS-CoV-2 neutralizing antibody responses are more robust in patients with severe disease. Emerg. Microbes Infect..

[15] Thieme C.J., Anft M., Paniskaki K., Blazquez-Navarro A., Doevelaar A., Seiberto F.S., Hoelzer B., Konik M.J., Moritz Berger M., Brenner T. (2020). Robust T cell response towards spike, membrane, and nucleocapsid SARS-CoV-2proteins is not associated with recovery in critical COVID-19 patients. Cell Rep. Med..

[16] Weiskopf D., Schmitz K.S., Raadsen M.P., Grifoni A., Okba N.M.A., Henrik Endeman H., van den Akker J.P.C., Molenkamp R., Koopmans M.P.G., van Gorp E.C.M. (2020). Phenotype and kinetics of SARS-CoV-2-specific T cells in COVID-19 patients with acute respiratory distress syndrome. Sci. Immunol..

[17] Grifoni A., Weiskopf D., Ramirez S.I., Mateus J., Dan J.M., Rydyznski Moderbacher C., Rawlings S.A., Sutherland A., Premkumar L., Jadi R.S. (2020). Targets of T Cell Responses to SARS-CoV-2 Coronavirus in Humans with COVID-19 Disease and Unexposed Individuals. Cell.

[18] Dan J.M., Mateus J., Kato Y., Hastie K.M., Yu E.D., Faliti C.E., Grifoni A., Ramirez S.I., Haupt S., Frazier A. (2021). Immunological memory to SARS-CoV-2 assessed for up to 8 months after infection. Science.

[19] Caballero-Marcos A., Salcedo M., Alonso-Fernández R., Rodríguez-Perálvarez M., Olmedo M., Graus Morales J., Cuervas-Mons V., Cachero A., Loinaz-Segurola C., Iñarrairaegui M. (2021). Changes in humoral immune response after SARS-CoV-2 infection in liver transplant recipients compared to immunocompetent patients. Am. J. Transplant..

[20] Thieme C.J., Anft M., Paniskaki K., Blazquez-Navarro A., Doevelaar A., Seibert F.S., Hoelzer B., Konik M.J., Meister T.L., Pfaender S. (2021). The Magnitude and Functionality of SARS-CoV-2 Reactive Cellular and Humoral Immunity in Transplant Population Is Similar to the General Population Despite Immunosuppression. Transplantation.

[21] Percivalle E., Cambiè G., Cassaniti I., Vecchio Nepita E., Maserati R., Ferrari A., Di Martino R., Isernia P., Mojoli F., Bruno R. (2020). Prevalence of SARS-CoV-2 specific neutralising antibodies in blood donors from the Lodi Red Zone in Lombardy, Italy, as at 06 April 2020. Eurosurveill.

[22] Li C.K., Wu H., Yan H., Ma S., Wang L., Zhang M., Tang X., Temperton N.J., Weiss R.A., Brenchley J.M. (2008). T cell responses to whole SARS coronavirus in humans. J. Immunol..

[23] Favà A., Donadeu L., Sabé N., Pernin V., González-Costello J., Lladó L., Meneghini M., Charmetant X., García-Romero E., Cachero A. (2021). SARS-CoV-2-Specific serological and functional T cell immune responses during acute and early COVID-19 convalescence in solid organ transplant patients. Am. J. Transplant..

[24] Williamson E.J., Walker A.J., Bhaskaran K., Bacon S., Bates C., Morton C.E., Curtis H.J., Mehrkar A., Evans D., Inglesby P. (2020). Factors associated with COVID-19-related death using OpenSAFELY. Nature.

[25] Favà A., Cucchiari D., Montero N., Toapanta N., Centellas F.J., Vila-Santandreu A., Coloma A., Meneghini M., Manonelles A., Sellarés J. (2020). Clinical characteristics and risk factors for severe COVID-19 in hospitalized kidney transplant recipients: A multicentric cohort study. Am. J. Transplant..

[26] Alberici F., Delbarba E., Manenti C., Econimo L., Francesca Valerio F., Pola A., Maffei C., Possenti S., Zambetti N., Moscato M. (2020). A single center observational study of the clinical characteristics and short-term outcome of 20 kidney transplant patients admitted for SARS-CoV2 pneumonia. Kidney Int..

[27] Coll E., Fernandez-Ruiz M., Padilla M., Moreso F., Hernández-Vicente A., Yañez I., Molina M., Vázquez-Sánchez T., Crespo M., Facund C. (2021). COVID-19 in Solid Organ Transplant Recipients in Spain throughout 2020: Catching the Wave?. Transplantation.

[28] Grupper A., Rabinowich L., Schwartz D., Schwartz I.F., Ben-Yehoyada M., Shashar M., Katchman E., Halperin T., Turner D., Goykhman Y. (2021). Reduced humoral response to mRNA SARS-CoV-2 BNT162b2 vaccine in kidney transplant recipients without prior exposure to the virus. Am. J. Transplant..

[29] Marinaki S., Adamopoulos S., Degiannis D., Roussos S., Pavlopoulou I.D., Hatzakis A., Boletis I.N. (2021). Immunogenicity of SARS-CoV-2 BNT162b2 vaccine in solid organ transplant recipients. Am. J. Transplant..

[30] Bertrand D., Hamzaoui M., Lemée V., Lamulle J., Hanoy M., Laurent C., Lebourg L., Etienne I., Lemoine M., Le Roy F. (2021). Antibody and T Cell Response to SARS-CoV-2 Messenger RNA BNT162b2 Vaccine in Kidney Transplant Recipients and Hemodialysis Patients. J. Am. Soc. Nephrol..

[31] Kamar N., Abravanel F., Marion O., Couat C., Izopet J., Del Bello A. (2021). Three Doses of an mRNA Covid-19 Vaccine in Solid-Organ Transplant Recipients. N. Engl. J. Med..

[32] Stumpf J., Tonnus W., Paliege A., Rettig R., Steglich A., Gembardt F., Kessel F., Kröger H., Arndt P., Sradnick J. (2021). Cellular and Humoral Immune Responses after Three Doses of BNT162b2 mRNA SARS-Cov-2 Vaccine in Kidney Transplant. Transplantation.

[33] Cavagna L., Seminare E., Zanframundo G., Gregorini M., Di Matteo A., Rampino T., Montecucco C., Pelenghi S., Cattadori B., Pattonieri E.F. (2020). Calcineurin Inhibitor-Based Immunosuppression and COVID-19: Results from a Multidisciplinary Cohort of Patients in Northern Italy. Microorganisms.

[34] Rovida F., Cassaniti I., Percivalle E., Sarasini A., Paolucci S., Klersy C., Cutti S., Novelli V., Marena C., Luzzaro F. (2021). Incidence of SARS-CoV-2 infection in health care workers from Northern Italy based on antibody status: Immune protection from secondary infection—A retrospective observational case-control study. Int. J. Infect. Dis..

[35] OMurchu E., Byrne P., Carty P.G., De Gascun C., Keogan M., O’Neill M., Harrington P., Ryan M. (2021). Quantifying the risk of SARS-CoV-2 reinfection over time. Rev. Med. Virol..

