# Supplementary material for: Robust and Persistent B- and T-Cell Responses after COVID-19 in Immunocompetent and Solid Organ Transplant Recipient Patients
Source: Viruses. 2021 Nov 11;13(11):2261. doi: 10.3390/v13112261 (PMC8621286; doi:10.3390/v13112261)
Supplement: Supplementary file 1 [file viruses-13-02261-s001.zip › viruses-1437645-supplementary.pdf]

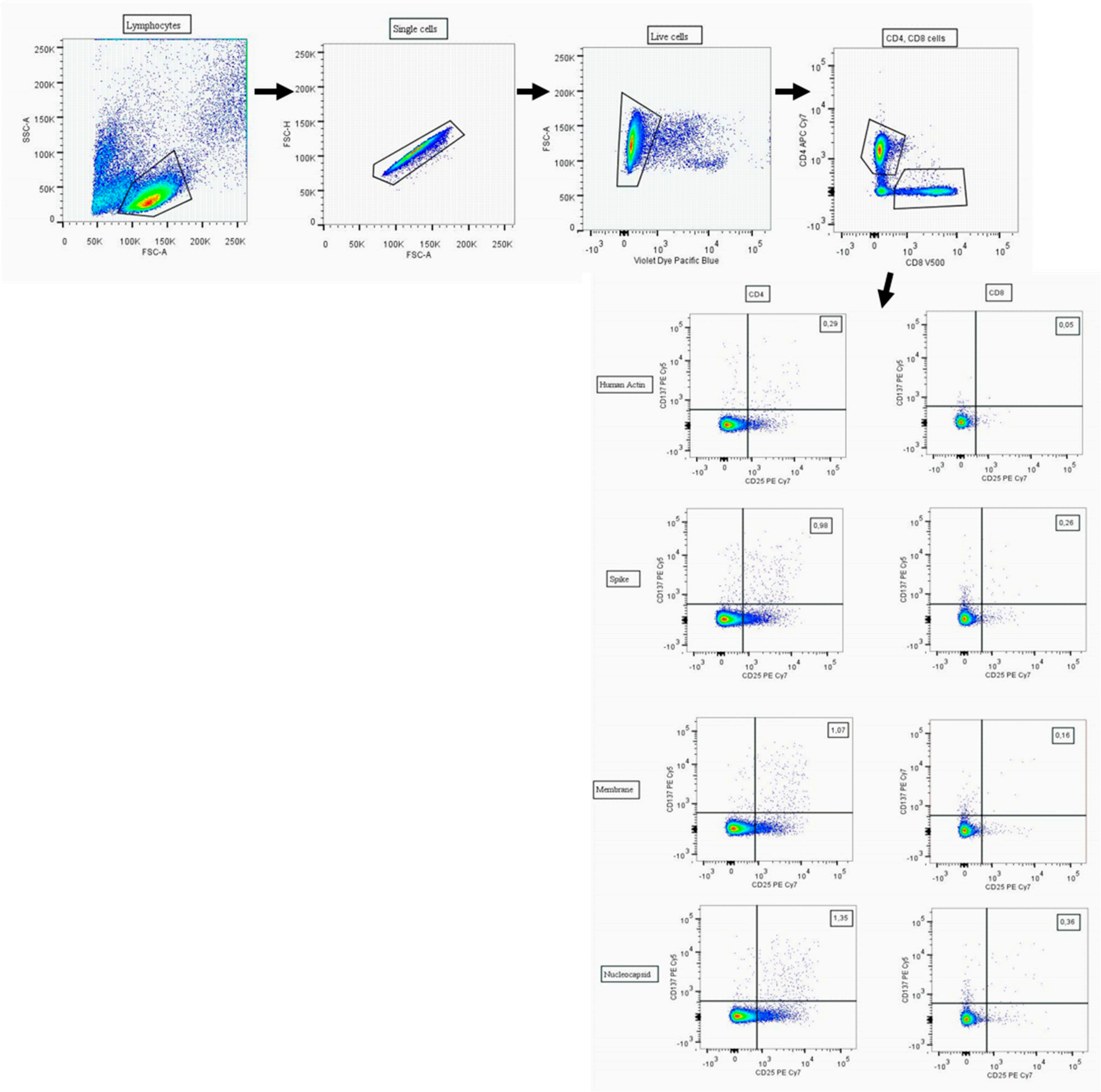

Figure S1. Activation Induction Marker Phenotyping Flow Cytometry. Representative gating of antigen specific effector CD4^{+} and CD8^{+} T-cells from post COVID-19 immunocompetent patients PBMC after stimulation with Spike, Membrane, Nucleocapsid and Human actin peptide pools. Briefly, lymphocytes cells were gated out of all events followed by subsequent singlet gating. Live cells are gated as Pacif Blue and cells were then gated as CD4 APC Cy7^{+} and CD8 V500^{+}. T-cells were further subdivided into CD4^{+} CD137 PECy5^{+} CD25 PECy7^{+} and CD8^{+} CD137 PECy5^{+} CD25 PECy7^{+} population.

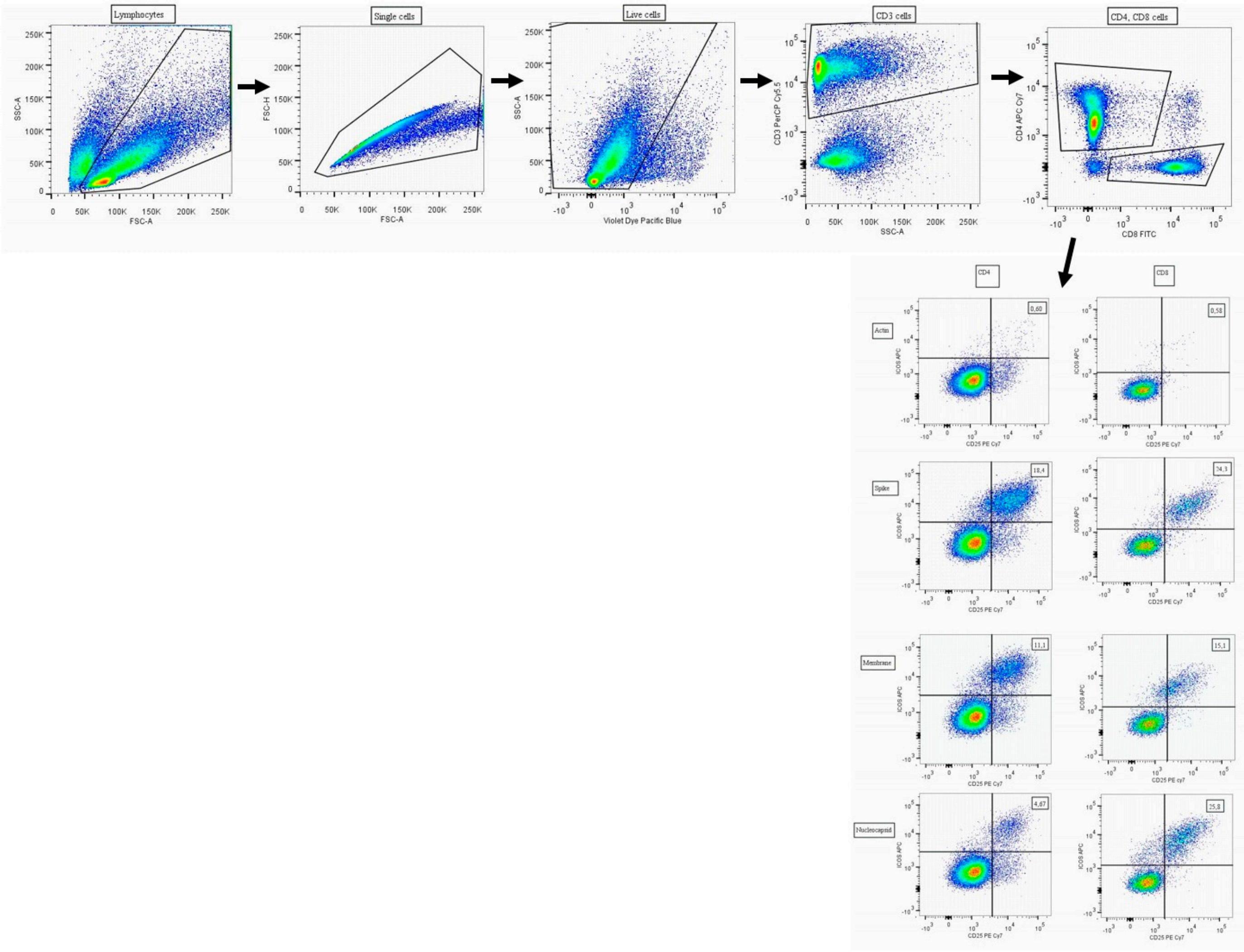

Figure S2. Lymphoproliferation Phenotyping Flow Cytometry. Representative gating of antigen specific memory CD4$^+$ and CD8$^+$ T-cells from post COVID-19 immunocompetent patients PBMC after stimulation with Spike, Membrane, Nucleocapsid and Human actin peptide pools. Briefly, lymphocytes cells were gated out of all events followed by subsequent singlet gating. Live cells are gated as Pacif Blue and cells were then gated as CD3 PERCP5.5$^+$. T cells were further subdivided into CD4 APC Cy7$^+$ and CD8 FITC$^+$ population. T cells were defined as CD4$^+$ ICOS APC$^+$ CD25 PECy7$^+$ and CD8$^+$ ICOS APC$^+$ CD25 PECy7$^+$.

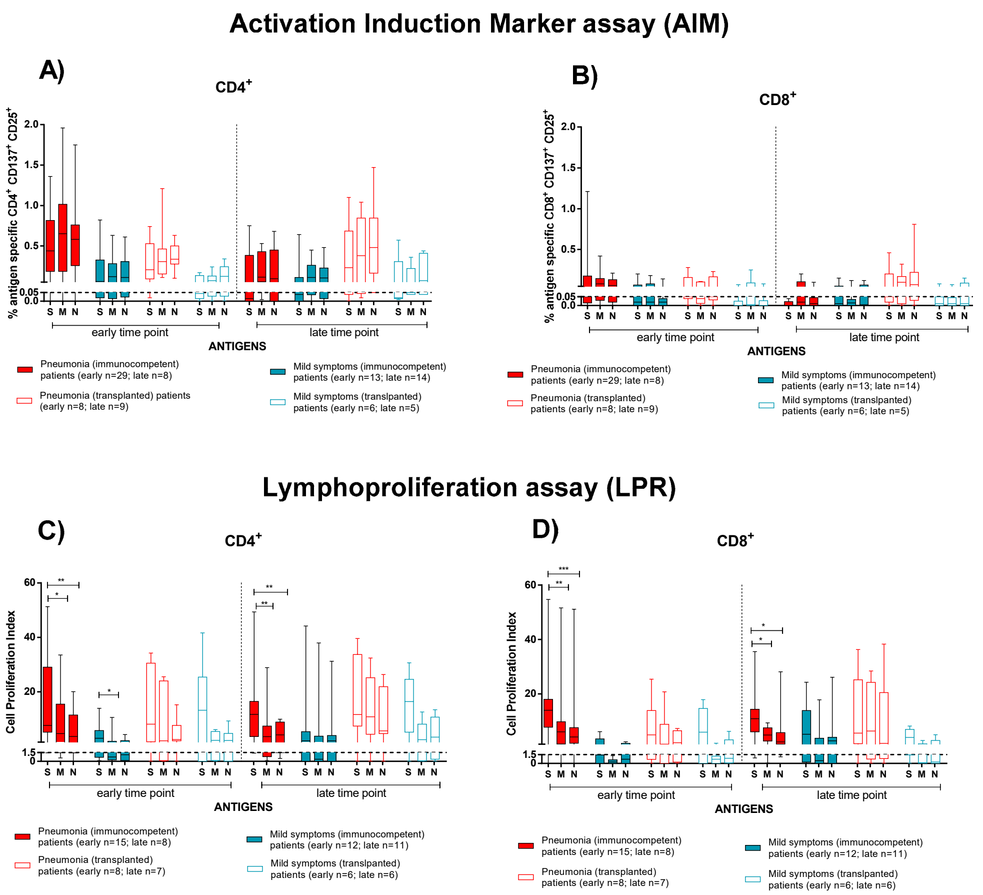

Figure S3. The T-cell dominant specificity after stimulation with Spike (S), Membrane (M) and Nucleocapsid (N) peptide pools in immunocompetent (IC) and transplanted (TX) patients with pneumonia or mild symptoms at early and late time points. A) SARS-CoV-2-specific $CD4^+$ and B) $CD8^+$ T-cells (rapid Activation Induced Marker assay); C) $CD4^+$ T Cell Proliferation Index; D) $CD8^+$ T-Cell Proliferation Index.

Early time point: patients with pneumonia, IC median 58 (range 45-100); TX 60 (30-62) days after infection; patients with mild symptoms, IC 48 (30-100); TX 54 (26-75) days after infection.

Late time point: patients with pneumonia, IC median 212 (range 186-400); TX 233 (164-309) days after infection; patients with mild symptoms, IC 192 (150-306); TX 167 (150-207) days after infection.

* $p < 0.05$; ** $p < 0.01$; *** $p < 0.001$.
